# The Community Health Response Team: a culturally and linguistically tailored community response to COVID-19 addressing barriers to testing and vaccinations for refugee, immigrant and migrant communities in Atlanta, Georgia

**DOI:** 10.3389/fpubh.2024.1362705

**Published:** 2024-02-23

**Authors:** Feven Bekele, Kimberly Yu, Sam Archbold, Erin Mann, Elizabeth Dawson-Hahn, Omar Aziz

**Affiliations:** ^1^International Rescue Committee, Atlanta, GA, United States; ^2^National Resource Center for Refugee, Immigrants, and Migrants, Center for Global Health and Social Responsibility, University of Minnesota, Minneapolis, MN, United States; ^3^Community Organized Relief Effort, Atlanta, GA, United States; ^4^General Pediatrics, University of Washington, Seattle, WA, United States

**Keywords:** refugees, immigrants, migrants, Community Health, vaccines, health promotion, minority health and health disparities

## Abstract

The International Rescue Committee (IRC) in Atlanta and Community Organized Relief Effort (CORE) established a Community Health Response Team in May 2020. The team members represented refugee, immigrant and migrant populations and had expertise in health care and public health. These 18 individuals were recruited from IRC Atlanta's Career Development program, had a variety of backgrounds and spoke 20 languages. They implemented a community-centered COVID-response intervention model of pairing education and outreach efforts with testing and vaccination clinics. Due to their team makeup, the Community Health Response Team conducted tailored outreach and education that was culturally and linguistically congruent with their target communities. They administered over 16,000 COVID-19 tests at mobile community sites within the first 6 months. Once COVID-19 vaccinations were available, the Community Health Response Team coordinated a total of 834 vaccination events in communities with a high number of refugees and in partnership with refugee- and immigrant-trusted community-based organizations, resulting in 31,888 vaccinations. Hiring staff from refugee, immigrant and migrant populations created a sustainable staffing model. Also, embedding culturally specific strategies in their model of pairing education and outreach led to long-term relationships and greater trust with community members. This approach of engaging and empowering community members to create tailored public health responses should serve as guidance for future public health campaigns.

## 1 Introduction and context

Historically, refugees face poorer health outcomes than host populations (1). When the COVID-19 pandemic began, International Rescue Committee's (IRC) Atlanta office and Community Organized Relief Effort (CORE) initiated a pandemic response effort for the communities they serve. The primary communities that IRC Atlanta and CORE serve are refugee, immigrant and migrant communities (RIM). The pandemic response effort initially focused on Clarkston, often described as “the most diverse square mile in America” (2) where the city has welcomed over 60,000 refugees since 1980 (3). As IRC Atlanta and CORE's partnership was established, the response effort expanded to larger DeKalb County in Atlanta with a focus on areas with more RIM communities. DeKalb County's population of 762,820 in 2022, with foreign born people making up 16.4% of the population (4).

With support and funding from Georgia Department of Public Health and the DeKalb County Board of Health, IRC Atlanta and CORE established a Community Health Response Team. The team was members of refugee, immigrant, and migrant populations with expertise and experience in health care and public health. The National Resource Center for Refugees, Immigrants, and Migrants (NRC-RIM) provided technical assistance to the IRC Atlanta office in carrying out various COVID-19 response work. The approach to building this team, responding to community health needs, and its resulting strength in trust-building with newcomers to the United States is described below as an example for future models for public health programs and responses to public health emergencies.

## 2 Key programmatic elements

IRC Atlanta implemented a COVID-response model involving a community-centered COVID response team. The intervention model of pairing education and outreach efforts with vaccination clinics effectively increased vaccination rates of the target community, and has potential applications for future public health emergencies.

### 2.1 Developing a community health response team

In May 2020, IRC Atlanta and CORE initiated a pandemic response for the refugee, immigrant and migrant communities they serve throughout the DeKalb County area. To serve this population, a team was hired to educate the community about COVID-19, disease prevention strategies, how to isolate and quarantine, as well as understanding and accessing COVID-19 testing. This partnership relied on the trust that IRC had already built with refugee communities and the operational resources that CORE had in place for COVID-19 testing.

**Figure 1:**
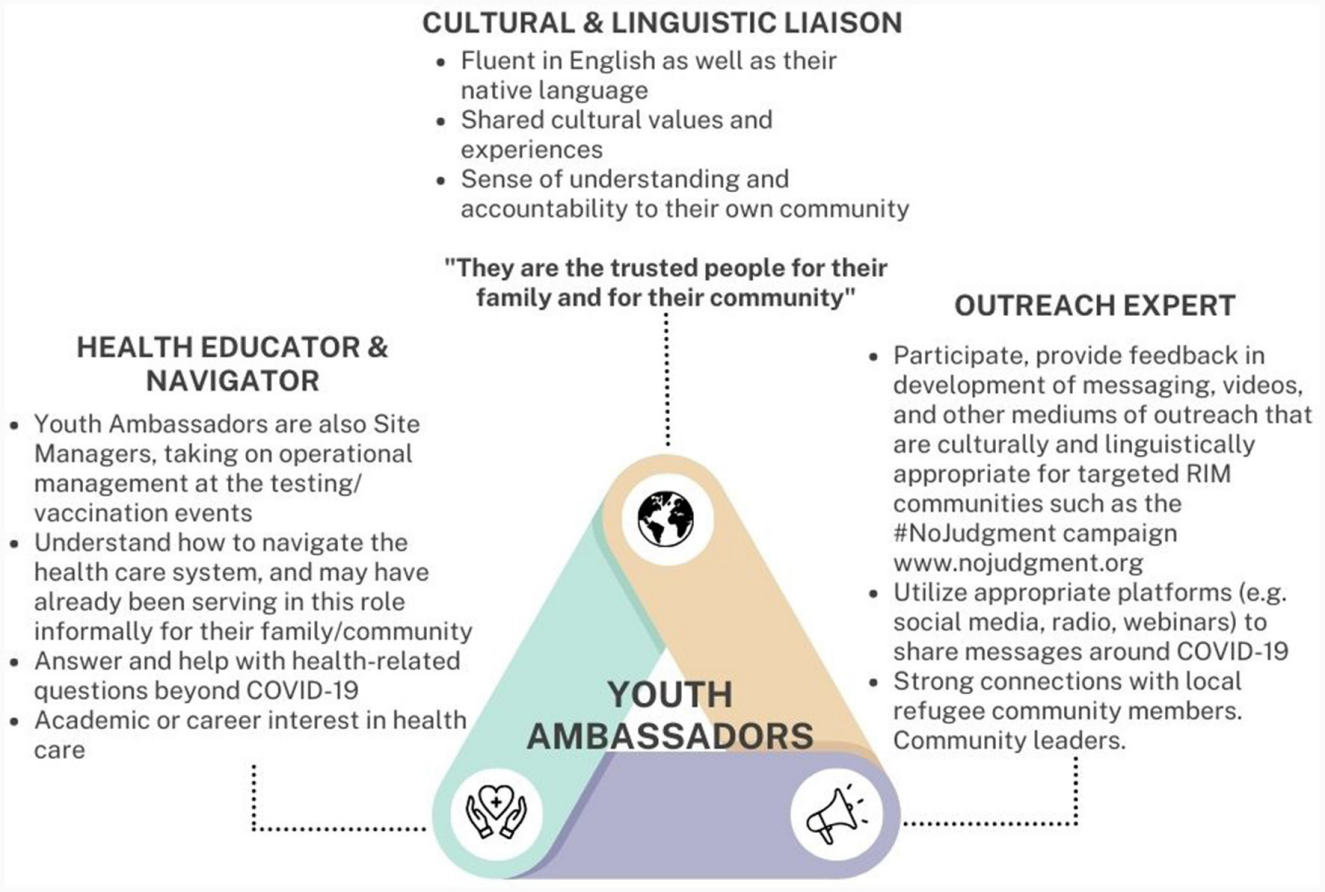
Youth Ambassadors—a team member with unique skills and role in response effort.

The response team was strategically hired through IRC Atlanta's Career Development program. Because recruitment efforts targeted those looking for careers in science, technology, engineering, and mathematics (STEM), nearly all members of the Community Health Response Team had degrees or work experience in STEM fields. The hired team consisted of 18 multilingual individuals that collectively spoke 20 different languages including Arabic, Amharic, Burmese, French, Keren, Lingala, Oromo, Spanish, Swahili (Kiswahili), and Zulu. This method of recruitment ensured that staff not only had linguistic congruence with the communities served, but also similar lived and cultural experiences. Among the 18 staff members were three Youth Ambassadors (Figure 1) that were identified as recent high school graduates pursuing a higher education degree in public health or health care and had a unique role as young people serving as cultural and linguistic liaisons for their communities.

All members of the Community Health Response Team were onboarded and trained as full-time staff, shadowed existing COVID-19 testing and vaccination clinics, additionally trained in the following topics:

- Protocols on safely collecting and entering personal information from testing and vaccination patients.
- Health Insurance Portability and Accountability Act (HIPAA) compliance training.
- Health education and health promotion trainings on COVID-19, and other relevant health issues with considerations for potential cultural concerns of the RIM community.
- Blood-born pathogen training.
- Protocol for testing kits including updated trainings when needed.
- Standard operating procedures and clinical requirements for clinic set up at various sites.

This team was developed to be able to respond quickly and effectively to community needs around COVID-19, as well as protocol or policy changes around testing and vaccination through team communication and the ability to communicate with community partners. Recognizing that strategies that serve the majority of the population in the United States would not be effective for the refugee communities, IRC Atlanta developed a team that could effectively conduct outreach, educate and build relationships with RIM communities during a public health emergency.

### 2.2 Developing a model for testing and vaccination

**Figure 2:**
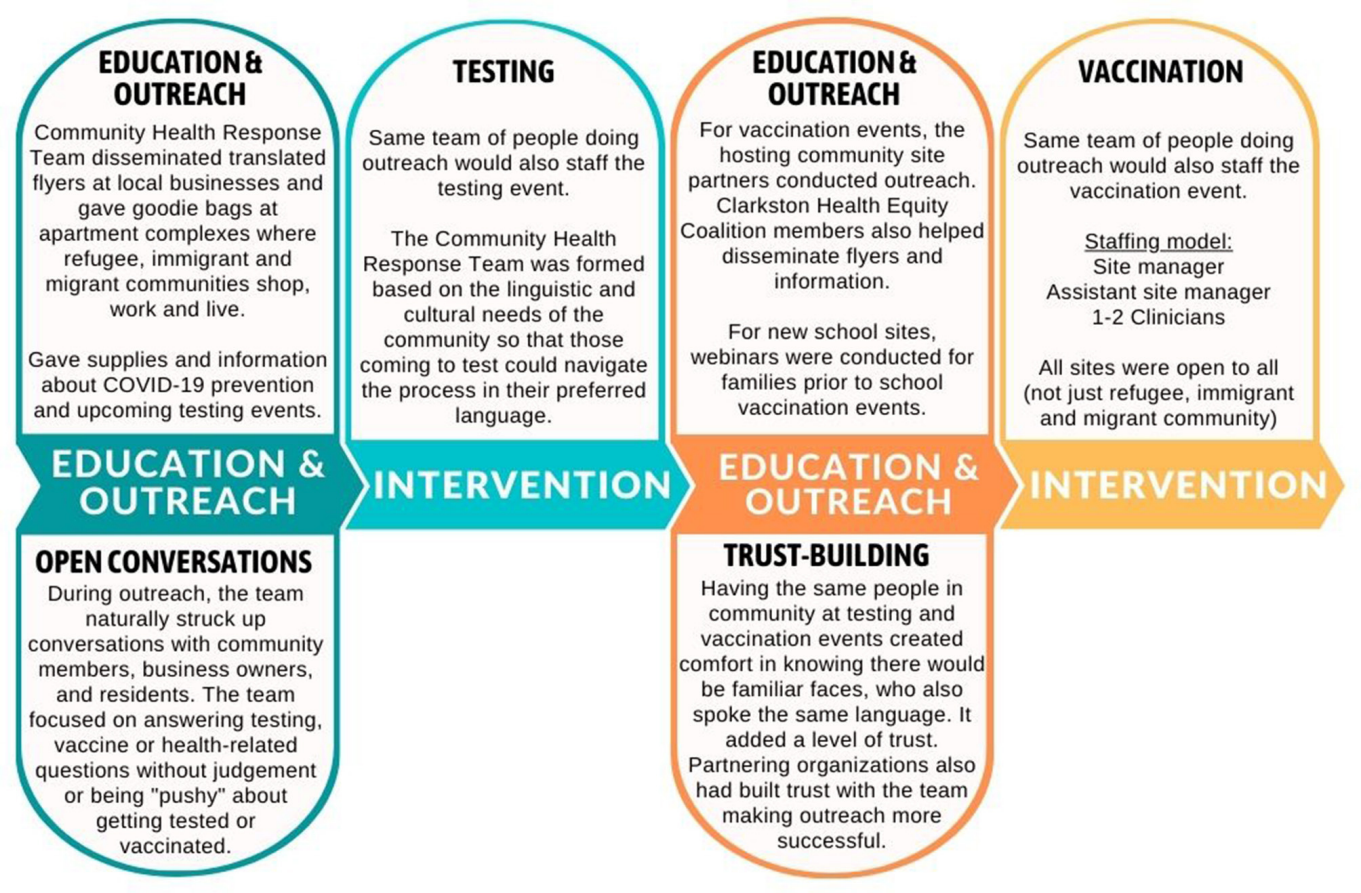
Education and outreach before intervention—model for COVID-19 testing and vaccination.

The Community Health Response Team understood the barriers refugee, immigrant and migrant communities face in trusting new government institutions, navigating a new language and health system, and accessing health care. So, COVID-19 testing services were only offered after education and outreach was conducted as shown in Figure 2. The team knew that, without the education and outreach prior to the clinical services, fewer people would get tested and vaccinated.

Although some outreach was conducted virtually (i.e., text/calls and webinars), the Community Health Response Team also did much of its work in-person to garner trust. When having in-person conversations, the team always worked in groups for personal safety and remained in contact with each other during outreach campaigns. They also followed protocol as determined by the Centers for Disease Control and Prevention regarding social distancing, sanitizing and personal protective equipment (or PPE).

To ensure that testing and vaccination sites were established in the areas of DeKalb County with the most need, CORE and IRC Atlanta utilized the following criteria:

- Identifying census tracts that had high Social Vulnerability Index (5) values.
- Reviewing data around hospital bed utilization or “hot spots” with high COVID-19 infection rates.
- Reviewing Georgia Department of Public Health data around regions in the state with low vaccine uptake.
- Anecdotal information from partners as shared during community meetings such as the Clarkston Health Equity Coalition.
- Assessing community site readiness to host clinics in terms of capacity of space and clinical operation requirements.

Once COVID-19 vaccines were approved in April 2021, the testing sites were converted to vaccination sites. The IRC Atlanta and CORE staff in DeKalb County shadowed the Fulton County CORE team, which had begun holding mobile vaccination clinics in February 2021. To operationally prepare for vaccinating, they learned how to set up a site, manage clinic flow and what to communicate to patients. The team continued to grow and streamline operations and, in July 2021, it divided into three groups to cover more of DeKalb County.

As the reach of the Community Health Response team grew, they continued to be responsive to community needs. This included assessing language needs and cultural concerns prior to testing or vaccination during education and outreach efforts in the community. Over time, the Community Health Response team adjusted clinics based on community needs such as expanding clinic hours outside of standard working hours, and sites with strong community presence hosting repeated and regular clinics.

### 2.3 Tailoring outreach strategies

The Community Health Response Team tailored its strategies to address each refugee, immigrant, and migrant community's specific barriers to testing and vaccination.

Some strategies used to provide education and allay concerns of various refugee, immigrant and migrant communities were:

1. Calling IRC Atlanta clients to talk one on one about vaccination, help with scheduling, and answering any questions.
2. Holding webinars on health topics with community experts and religious leaders (e.g., Imam and pastor) to answer any community questions and concerns in their native language and deliver information through trusted and respected sources.
3. Hosting Facebook Live events with community members to share personal vaccination stories with the larger community and help alleviate anxiety around vaccination.

In addition, partner organizations were able to conduct outreach through the preferred channels of their community members. Some examples include, texting, calling, social media (e.g., Facebook and WhatsApp), and in-person conversations at community events.

Multilingual flyers were widely used to promote vaccination using three distribution methods. First, translated flyers about upcoming vaccination clinics and current eligibility requirements were disseminated through all IRC partners and staff via weekly emails. They included QR codes and URLs for easy access to event information. Especially when eligibility requirements were changing frequently, it was important to provide updated information. Second, flyers were posted at key local businesses with additional copies given to the owners to distribute. Finally, knowing that certain refugee communities live in specific apartment complexes, the response team worked with leasing offices to distribute vaccination information in “goodie bags” for residents. This also created opportunities for in-person conversations and gaining trust with residents.

To address transportation barriers experienced by RIM communities (6), the team contracted with ride-share services to provide transportation to and from vaccination events.

Beyond the operational support of the testing clinics themselves, the Community Health Response Team was available to provide language support for public health contact tracing conducted when people tested positive for COVID-19. This effort of continued communication strengthened RIM community trust with the team, and as a result, they could better address misinformation and any fears that community members had around COVID-19.

All activities conducted by the Community Health Response Team included clearly visible branding with the IRC Atlanta and CORE logos, because of the existing positive reputation that these organizations have in the RIM community. This reputation helped increase the potential for people to trust the information that the team provided to address existing misinformation or fears around COVID-19. For example, IRC Atlanta delivers furniture and other supplies to refugees when they find housing, so community members have an existing positive association with the IRC Atlanta truck and know that the organization provides resources and support. Because of this reputation, this truck with large IRC Atlanta branding was present at every testing and vaccination site. As clinics continued, branding included IRC Atlanta, CORE, and DeKalb Public Health, expanding that network of trusted organizations through the partnerships that established the Community Health Response Team.

## 3 Impact metrics

The Community Health Response Team successfully reached many refugees, immigrants, and migrants in DeKalb County through COVID-19 outreach, education, testing, and vaccination.

From April 2021 to August 2022, more than 3,500 calls were made or 250 families were contacted through IRC Atlanta's list of clients from the last 5 years. These calls served to conduct health and wellness checks, share community resources, provide education on COVID-19 prevention, and check in on their vaccination status. With the influx of new arrivals from Afghanistan, many of those reached were newly arrived refugees, and for all contacts, English was not the preferred language.

Although conversations from phone calls and in-person outreach conducted before every testing and vaccination event, the Community Health Response Team also conducted digital outreach in the form of weekly emails with education and translated materials to over 160 partners and entities, as well as holding over 15 Facebook Live and webinar events from 2021 to 2022.

Outreach efforts of the Community Health Response Team were strengthened by the existing community trust in IRC Atlanta as the resettlement agency that had already supported much of the RIM community in the region. Because of the outreach and education conducted, the team performed over 16,000 COVID-19 tests within the first 6 months of testing in mobile sites in the community (May 2020; August–December 2020).

Between April 2021 and November 2022, the Community Health Response Team hosted 834 total vaccination events in partnership with various community organizations and entities. These events included the following types:

- Pop-up clinics (e.g., health fairs).
- Faith-based events (e.g., mosque, temple, or church).
- Community center events (e.g., cultural centers or led by ethnic community-based organizations).
- School-based vaccination events.
- Cultural events (e.g., cultural holiday events or festivals).

**Figure 3:**
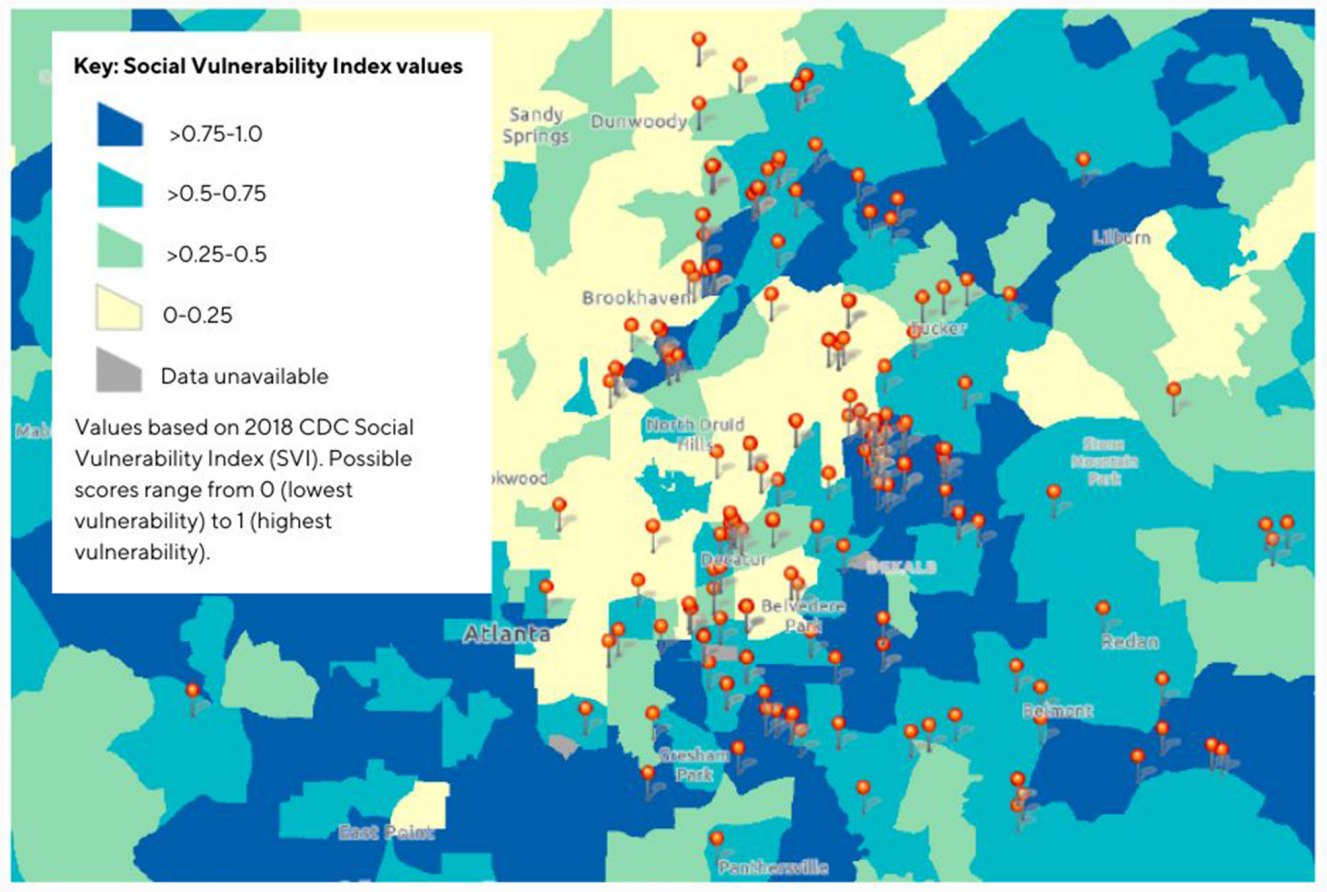
Map for Community Health Response Team* COVID-19 Mobile vaccination events May 2021–November 2022 and corresponding Social Vulnerability Index values. 2018 CDC SVI Fact sheet: https://www.atsdr.cdc.gov/placeandhealth/svi/fact_sheet/fact_sheet.html. *A partnership program of International Rescue Committee of Atlanta and Community Organized Relief Effort.

The team held vaccination events in locations (Figure 3) that would best serve those in need of the tailored response provided. As shown in Figure 3, most of the vaccination events were held in census tracts with high Social Vulnerability Index (5) scores. The index measures 16 factors, including poverty, lack of vehicle access, and crowded housing, that indicate “social vulnerability.”

These events were the outcome of partnerships that were developed through the Clarkston Health Equity Coalition. This coalition was a result of the efforts of IRC Atlanta and CORE bringing together a task force to respond to the rapidly evolving COVID-19 information and public health recommendations in the early phases of the pandemic. These partnerships range from schools and religious centers to local businesses and community organizations. Through this coalition and other community relationships, there were 165 community partners that held testing and vaccination events since May 2020. In addition, about 45 community partners supported these efforts through outreach through various methods such as dissemination of flyers, social media, and word of mouth.

As a result of the 834 events held from April 2021 to November 2022, they were able to administer 31,888 vaccinations. The strategies and approaches of the Community Health Response Team contributed to the vaccination rate in the DeKalb area being 20% higher than comparable areas in Georgia (7). The impact of these targeted campaigns to test and vaccinate RIM communities is especially significant given the findings of Georgia State University's Prevention Research Center seeing poorer outcomes in every measure of health and welfare for refugees vs. non-refugees through a community needs assessment conducted in Clarkston, Georgia (8), thus indicating a greater need for these services.

## 4 Discussion

### 4.1 Keys to success

*Hiring staff from the refugee, immigrant and migrant community* set the foundation for a strong team with the skillset to appropriately respond to community needs. In a time where there were so many unknowns around COVID-19 and the nature of the disease, having trusted people to turn to when there were questions helped the community stay healthier and close gaps in health outcomes. Having shared lived experiences with the communities served gave the team insight into how to effectively build trusting relationships with people. This trust helped combat misinformation, fears, and stigma around COVID-19. Not only did hiring from the refugee, immigrant and migrant community strengthen the public health campaign to educate, test and vaccinate this community, but it also helped *build community capacity* to respond appropriately to meet their own needs during a public health emergency.

Among the team members, one unique type of staff role to highlight were the Youth Ambassadors, who were able to serve as a bridge between their home culture and the culture and systems of the United States (Figure 1). To hire these members, IRC Atlanta identified and recruited young community leaders who may have already been serving this role informally within their communities, had the linguistic and cultural knowledge, and also had interest in health care (Figure 1).

The Community Health Response Team maintained the same core staffing throughout the COVID-19 pandemic, even when future funding was uncertain. Team members already had connections to their communities and interest in public health; they were not only looking for experience in the field but also felt accountable to the communities they were serving. With this *sustainable staffing model*, the same individuals navigated throughout all phases of the COVID-19 pandemic, internal communication was clear, and changes could be made quickly. For example, when the bivalent booster replaced the monovalent booster in one day, the team quickly addressed fears that the original booster had been discontinued because it was dangerous or had expired.

In developing these approaches, the Community Health Response Team recognized that inequities in COVID-19 vaccination rates resulted from many factors, including lack of knowledge around the vaccines and how to access them. To address this, they *paired educational outreach with every testing or vaccination event*. This allowed for community members to voice their concerns openly to people who spoke their language and had shared cultural knowledge.

Recognizing the diversity within the refugee, immigrant and migrant community, the Community Health Response Team made efforts to implement *culturally and community specific strategies*. These included: partnering with trusted community organizations and cultural and religious leaders, using communication methods preferred by each community (e.g., WhatsApp or Facebook), attending cultural events or holidays, and ensuring materials are linguistically and culturally appropriate for the community. For example, partnering with Imams in working with the Muslim community to address questions around the Halal status of COVID-19 vaccines. From this partnership, the team created videos in seven languages to address this concern.

The team valued *building long-term relationships* with the community. By having familiar faces at most events, they built trust as people saw the same team members conducting education, testing, vaccinations and contact tracing interpretation. Responding to community feedback obtained through the relationships built by the Community Health Response Team, CORE funded additional staff to support needs beyond COVID-19 testing and vaccination (e.g., providing food for those in quarantine). Also, IRC Atlanta and CORE were able to work as parts of a concerted effort by fostering partnerships with the more than 30 member organizations of the Clarkston Health Equity Coalition as well as other organizations. Having a continued presence and growing these working partnerships with other organizations, IRC and CORE have been able to more effectively work in a concerted effort to address refugee, immigrant and migrant community needs around COVID-19 and beyond.

Cutting across these keys to success is *building trust* with community members. Recognizing that asking community members to trust the team with supporting an aspect of their health, the Community Health Response Team's strategies and approaches were always rooted in listening to community voices and valuing their trust.

### 4.2 Challenges addressed

The main barriers to vaccination that the refugee, immigrant and migrant community faces are around language, culture, and transportation. With staff and resources to translate materials, ensure their cultural relevance, and speak with community members in their preferred language, the Community Health Response Team effectively addressed language and cultural barriers to vaccination. The program also held clinics in locations frequented by RIM community and contracted with ride-share services to provide transportation to and from vaccination events for those that could not easily find transportation for themselves.

With public health campaigns and this type of community outreach being new to many refugee, immigrant and migrant community members, Community Health Response Team efforts to do outreach in the community often led to many people questioning why they were there, who was behind their efforts, and questions around vaccines. Being able to speak the same language as those community members and being understanding of their concerns without judgment, opened up a lot of conversations that helped address many community concerns and fears during an ever-changing public health emergency. The team was also able to rely on the positive reputation of IRC Atlanta and CORE while serving as educated experts taking the time to address questions, talking about trusted community leaders' (e.g., religious leaders) stances on vaccination, and sharing personal testimonials. Through these conversations the team was able to successfully recruit participants that would otherwise struggle to navigate mainstream or mass vaccination/testing sites.

### 4.3 Addressing future public health emergencies

The COVID-19 pandemic highlighted many inequities that DeKalb County's refugee, immigrant and migrant communities had already been experiencing. The creation of the Community Health Response Team and their approach to public health interventions demonstrated how public health responses can be tailored to meet the needs of these and other underserved groups. The process of developing a Community Health Response Team, establishing the Clarkston Health Equity Coalition, and slowly building community trust in public health and health systems, increased community capacity to respond to public health crises.

Since May 2020, when IRC Atlanta and CORE first established the Community Health Response Team, there has been a demonstrated need by community for increased public health presence and services. By 2021, Georgia Department of Public Health and the DeKalb County Board of Health funded and supported the expansion of the team's activities. Now many of CORE's efforts are focused not only on maintaining the network of partners developed during the height of COVID-19, but also on strengthening public health infrastructure beyond emergency funding. Partners in the Clarkston Health Equity Coalition include social services (e.g., Supplemental Nutrition Assistance Program or SNAP), community based organizations, health care services and more. In 2023, the efforts of the Community Health Response Team continue to support these efforts to provide and connect the RIM community to comprehensive health care and social services, in addition to annual vaccinations for COVID-19 and influenza.

Although the focus of the Community Health Response Team was around COVID-19 testing and vaccination, through conversations and relationships built with community members, the team helped patients navigate other health concerns and questions. There is potential in these existing relationships to quickly and effectively address a myriad of current and future public health issues beyond COVID-19. The model of creating tailored intervention strategies through engaging and empowering community members in the response effort should serve as guidance for future public health campaigns and programs.

## 5 Limitations

The authors acknowledge that content for this case study was primarily based on conversations and listening sessions with key leaders of the Community Health Response Team. Community partners and their perspectives were not included due to limited resources and capacity of the research team. Instead, the authors chose to reflect the nature of the community partnerships through growth of such partnerships and continued collaboration with the Community Health Response Team.

## Data availability statement

The raw data supporting the conclusions of this article will be made available by the authors, without undue reservation.

## Author contributions

FB: Writing – original draft, Writing – review & editing, Conceptualization, Project administration, Supervision. KY: Writing – original draft, Writing – review & editing, Project administration, Visualization. SA: Data curation, Writing – review & editing. EM: Conceptualization, Supervision, Writing – review & editing. ED-H: Conceptualization, Supervision, Writing – review & editing, Funding acquisition. OA: Supervision, Writing – review & editing.
